# Long-term trend of antibiotic use at public health care institutions in northwest China, 2012–20 —— a case study of Gansu Province

**DOI:** 10.1186/s12889-022-14944-6

**Published:** 2023-01-05

**Authors:** Wenxuan Cao, Hu Feng, Yongheng Ma, Defang Zhao, Xiaobin Hu

**Affiliations:** 1 School of Public Health, Lanzhou University, Lanzhou, 730000 China; 2 Division of Pharmaceutical Procurement, Gansu Public Resources Trading Center, Lanzhou, 730000 China

**Keywords:** Antibiotic use, Public hospitals, Primary health care centres, Quality indicators, China

## Abstract

**Background:**

Over the past 20 years, excessive antibiotic use has led to serious antimicrobial resistance (AMR) worldwide, and the phenomenon is particularly serious in China. To this end, the Chinese health sector took a series of measures to promote rational antibiotic use. In this study, to reveal the impact of policies on antibiotic use, we explored the long-term trend and patterns of antibiotic use at public health care institutions from 2012 to 2020 in northwest China, taking Gansu Province as an example.

**Methods:**

Antibiotic procurement data were obtained from the provincial centralized bidding procurement (CBP) platform between 2012 and 2020. Antibiotic use was quantified using the Anatomical Therapeutic Chemical (ATC)/defined daily doses (DDD) methodology and standardized using the DDD per 1000 inhabitants per day (DID). Twelve relevant quality indicators were calculated for comparison with the European Surveillance of Antimicrobial Consumption (ESAC) project monitoring results.

**Results:**

Total antibiotic use increased from 18.75 DID to 57.07 DID and then decreased to 19.11 DID, a turning point in 2014. The top three antibiotics used were J01C (beta-lactam antibacterials, penicillins), J01F (macrolides, lincosamides and streptogramins), and J01D (other beta-lactam antibacterials, cephalosporins), accounting for 45.15%, 31.40%, and 11.99% respectively. The oral antibiotics used were approximately 2.5 times the parenteral antibiotics, accounting for 71.81% and 28.19%, respectively. Different use preferences were shown in public hospitals and primary health care centres (PHCs), and the latter accounted for more than half of total use. The absolute use of all classes of antibiotics in Gansu is almost higher than any of the 31 European countries included in the ESAC, but the relative use of some focused antibiotics is lower than theirs.

**Conclusions:**

The intervention policies of the health department reduced antibiotic use in Gansu Province, but the proportion of broad-spectrum and parenteral antibiotics was still high. It is necessary to further improve the quality of antibiotic prescriptions and pay more attention to the rationality of antibiotic use in PHCs.

## Introduction

Over the past 20 years, global antibiotic consumption has increased by 65%, with the majority being dispensed from low- and middle-income countries (LMICs) [1]. With the widespread use of antibiotics worldwide, antimicrobial resistance (AMR) has become a global public health problem following bacterial infections, endangering human health and life, and causing an enormous health economic burden [2]. An important assessment published in 2015 showed that at least 700,000 people worldwide die of AMR infections every year. This number was expected to rise to 10 million by 2050 if the foregoing circumstance was not controlled, and the total loss of the global economy may exceed $US100 trillion [3]. However, this analysis included only six pathogens and the acknowledged real number may be much higher [4].

As a representative of developing countries, China ranks second in the world in human antibiotic use, only lower than India [5]. Research has shown that prescriptions approved by Chinese doctors have always been characterized by excessive and unreasonable antibiotic use, and the antibiotic use rate in outpatients was close to or more than 50% [6], which was higher than in other developing countries (40%-50%). However, as recommended by the World Health Organization (WHO), the reasonable range of this index was 20.0%-26.8% [7], and China was higher than this recommended value. In addition, due to the lack of continuous high-quality education and training for health providers, this phenomenon was more common at primary health care centres (PHCs). Some research results showed that the antibiotic use rate in rural areas of China was generally 50%-70% [8–10].

The Chinese government never gave up using policy intervention to regulate antibiotic use. As early as 2004, the former Ministry of Health issued 'Guiding principles for clinical application of antibiotics' [11], which systematically standardized antibiotic use and management in health care institutions for the first time. However, due to the lack of mandatory requirements, the implementation of different health care institutions were uneven, and antibiotic use did not achieve the desired effect. To promote rational antibiotic use, China established the National Centre for Antibacterial Surveillance in the next year [12], but it was not until 2010 that all provinces and cities carried out the work in a comprehensive manner. After that, a series of relevant strategies and guidelines had little effect. By August 2012, the 'Administrative Measures for Clinical Use of Antimicrobial Agents' were implemented nationwide in the form of an order from the Ministry of Health. The policy required health care institutions to establish hierarchical management measures, monitor and analyse the trend of clinical antibiotic use and bacterial resistance, and strengthen interventions in irrational medicine use [13]. These steps gradually promoted the normalization and refinement of antibiotic use and management [14].

In 2009, the Chinese government issued an essential medicines programmer and implemented a zero mark-up policy while establishing the provincial centralized bidding procurement (CBP) platform to ensure the supply of essential medicines [15], which provided a good channel for public health care institutions to purchase essential medicines. However, a systematic review revealed that despite the decline in the price of essential medicines, the impact of policy on antibiotic prescriptions in PHCs was very limited. Some results reported that number of antibiotic prescriptions had no statistically significant difference in PHCs before and after the policy [16].

The European Surveillance of Antimicrobial Consumption (ESAC) project recorded the information related to antibiotics in the sales records and reimbursement systems of 31 countries in Europe [17], which provided accurate data support to compare antibiotic use in China on an international level. The twelve quality indicators summarized by its scientific advisory board can be used to evaluate the potential unreasonable use of antibiotics in PHCs. This method has been proven to be effective in other studies [18]. This study collated antibiotic use data of Gansu Province from 2012 to 2020, analysed antibiotic use in different health care institutions and compared it to countries monitored by ESAC. This study aimed to (1) explore the long-term trend and mode of antibiotic consumption in northwest China, (2) reveal differences between this region and other countries and regions, and (3) provide clues and help decision-makers to formulate relevant policies and measures.

## Methods

### Study setting

Gansu is an underdeveloped province in northwest China. Its GDP was $US141.76 billion in 2020 —— the fifth lowest in the country, and the second-lowest in per capita GDP [19]. Since the beginning of the three-year new medical reform (2009–11), Gansu has included government-run medical and health institutions in the implementation of the national basic drug management approach and has achieved zero markups on drugs. By the end of 2012, the coverage rate of the essential medicines scheme reached 100% [20].

PHCs, in a broad sense, are medical institutions at the township (community) level and small medical and health service stations (village health offices, community health service stations, etc.). They are mainly responsible for providing comprehensive services such as public health services and the treatment of common and multimorbid diseases. China's primary health care institutions have been suffering from a shortage of doctors and low service capacity [21]. Due to the large rural population, small population density and large service radius, primary health care in Gansu is more difficult to carry out and of lower quality. Several relevant studies showed the phenomenon of excessive and unreasonable antibiotic use and serious AMR problems in Gansu, which were particularly prominent in PHCs [22–24].

### Data source

The data on antibiotic use in this study were extracted from the CPB platform of the Gansu Public Resources Trading Centre. As the only medicine procurement system dominated by the government, operated online and faced by almost all public health care institutions, the platform was first established in 2009 [25]. By 2020, the number of public health care institutions purchasing antibiotics through this platform reached 1900 in the province, including 272 public hospitals and 1510 PHCs (Table 1), accounting for 93.39% of the total number of public medical institutions in the province. We collected data related to antibiotics on the platform from 2012 to 2020, and its main record information included the general name, dosage form, specification, procurement unit, procurement quantity, etc.

**Table 1:** Number of public health care institutions covered by the CPB in Gansu, 2012–20

Year	public hospitals			Total number of hospitals	PHCs		Total number of PHCs	Others
	General hospitals	Traditional Chinese hospitals	Specialized hospitals		Urban PHCs	Rural PHCs		
2012	137	55	2	194	123	1248	1371	52
2013	142	66	7	215	144	1290	1434	78
2014	150	68	7	225	147	1271	1418	90
2015	152	71	8	231	162	1328	1490	95
2016	159	72	8	239	158	1318	1476	99
2017	165	72	15	252	162	1323	1485	101
2018	177	73	16	266	173	1339	1512	107
2019	178	74	19	271	182	1344	1526	110
2020	180	72	20	272	177	1333	1510	118

### Data management

After deleting a few data with incomplete information, the Anatomical Therapeutic Chemical (ATC) system developed by WHO was used to code and classify medicine, and then was used to conveniently screen the specific medicine classes related to the study. In this paper, all "J01" (antimicrobials for system use) in "J" (anti-infectives for system use) were included in the study. The dataset was managed using Microsoft Office Excel 2016 and computed using Stata (version 15.0), and charts were plotted using GraphPad Prism 9.

### Data analysis

The use of various antibiotics was calculated with the defined daily doses (DDD) as the minimum dose unit, and the total amount of purchases for all packaged units was expressed as DDDs and standardized with the DDD per 1000 inhabitants per day (DID). DDD, as the unit value of medication frequency analysis, represented the average maintenance dose per day in adults for its major therapeutic purposes; DDD equivalence per package (DPP) was an intermediate indicator for calculating total DDDs, and the calculation formula was DPP = (unit strength × pack size)/DDD, $DDDs= {\sum }_{i=1}^{n}{DPP}_{i}\times {N}_{i}$, where ${N}_{i}$ represents the number of packages of a certain product ($i$). The standardized medication intensity was calculated by DID, which indicated the DDD per 1000 inhabitants per day.

The number of inhabitants was represented by the average population every two years in the Gansu Statistics Yearbook [26]. According to these data, we found that approximately 60% of total outpatient visits were in hospitals and 40% were in PHCs. Outpatient visits by public health care institutions accounted for approximately 85% of all health care institutions [27]. Therefore, public hospitals shared approximately 51% of the total outpatient visits, and PHCs shared 34% in Gansu. We adjusted the share of antibiotic use in different health care institutions with the above data (assuming that patients chose health care institutions without crossing, that is, only choosing public or private health care institutions to visit).

### Comparison with ESAC monitoring results

The purchasing units were divided into public hospitals and PHCs according to their levels. The consumption and use preferences of various antibiotics in different institutions were calculated, and the results were compared with monitoring results in the ESAC project [28]. Eighteen use indicators were calculated to indicate total antibiotic use, which were respectively J01, J01A (tetracyclines), J01B (amphenicols), J01C, J01D, J01E (sulfonamides and trimethoprim), J01F, J01G (aminoglycoside antimicrobials), J01M (quinolone antibacterials), J01CA (broad-spectrum penicillins), J01CE (beta-lactamase sensitive penicillins), J01CR (combinations of penicillins), J01DB (first-generation cephalosporins), J01DC (second-generation cephalosporins), J01DD (third-generation cephalosporins), J01FA (macrolides), J01FF (lincosamides) and J01MA (fluoroquinolones). Twelve relevant quality indicators were calculated to compare the difference in antibiotic use between different PHCs, which were the use of J01, J01C, J01D, J01F, J01M, and the percentages of J01CE, J01CR, J01DD + J01DE (fourth-generation cephalosporins), J01MA in total volume, as well as the ratio of broad/narrow-spectrum antibiotic use and seasonal variations of J01 and J01M use.

## Results

### Overview of antibiotic use in Gansu, 2012–20

**Fig. 1:**
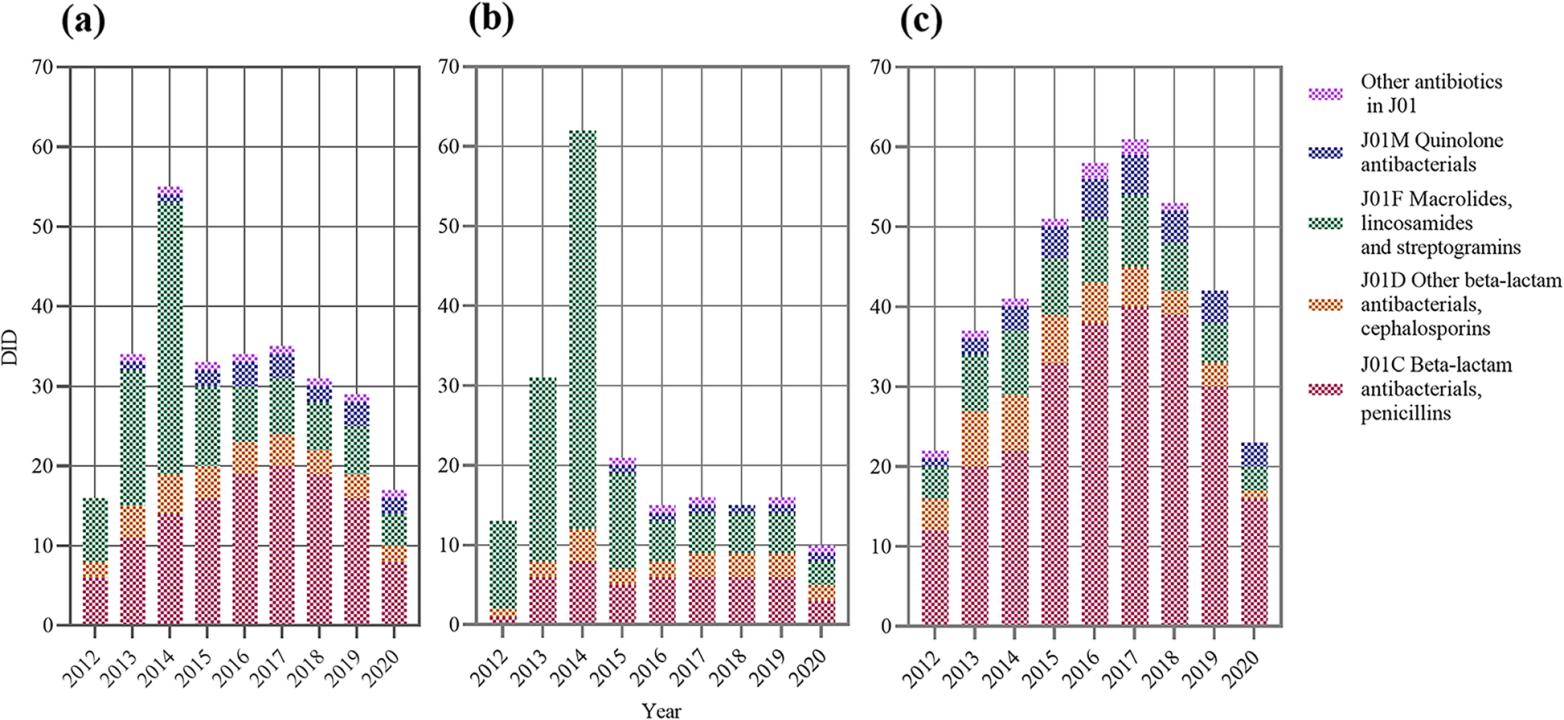
**a** Total use of antibiotics in Gansu, 2012–20. **b** Antibiotic use at public hospitals in Gansu, 2012–20. **c** Antibiotic use at PHCs in Gansu, 2012–20

The total antibiotic use in Gansu Province increased from 18.75 DID in 2012 to a peak of 57.07 DID in 2014, and then decreased to 19.11 DID in 2020. Consumption rose fastest in 2013 (95.97%) and fell fastest in 2015 (36.70%). Such a trend was mainly shaped by changes in J01F (Fig. 1). The changing trend of parenteral antibiotics was the same as that of total antibiotics and oral antibiotics peaked in 2017. The latter (23.55 DID, 71.81%) was approximately two times the former (10.33 DID, 28.19%) in average use.

**Fig. 2:**
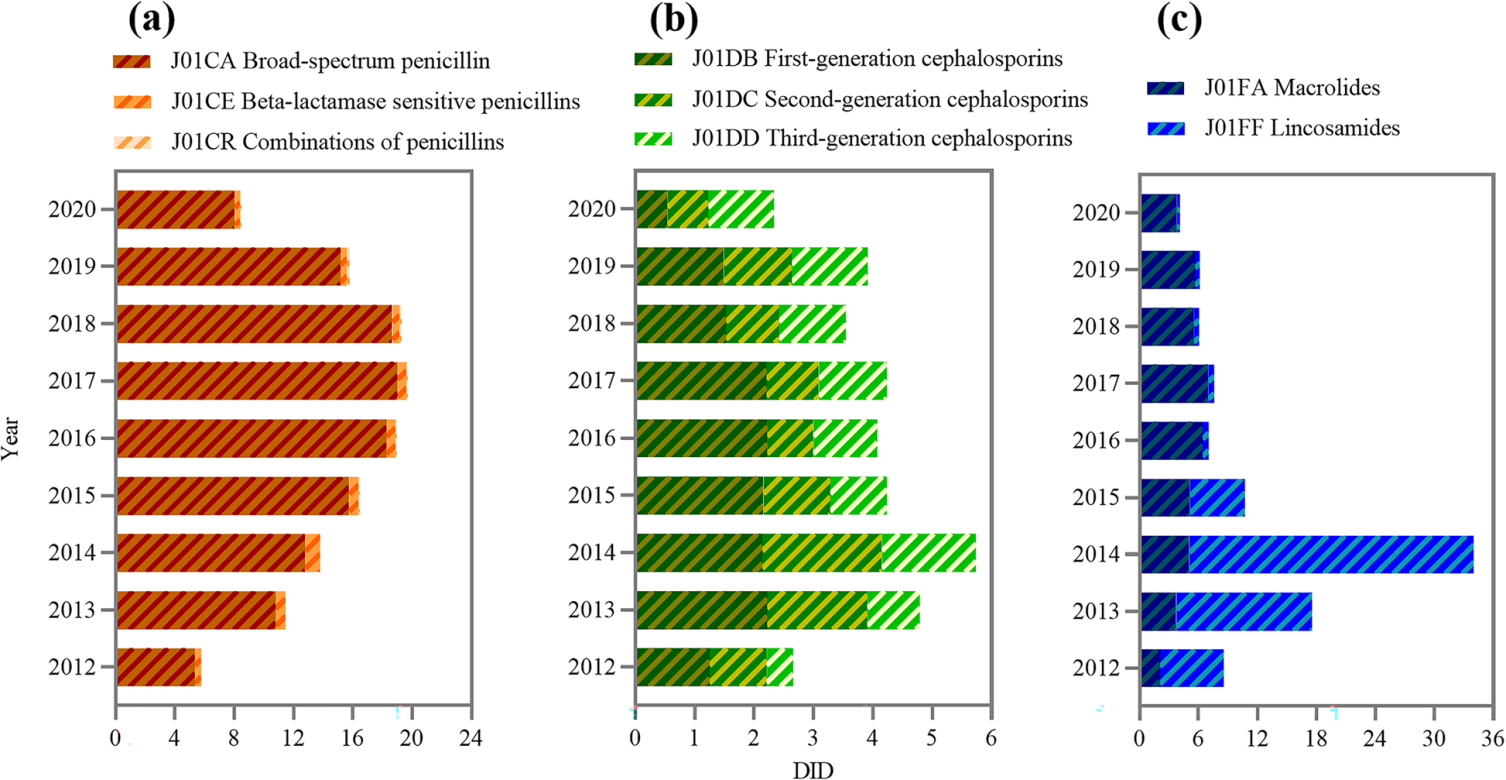
**a** J01C (beta-lactam antibacterials, penicillins) use in Gansu, 2012–20. **b** J01D (other beta-lactam antibacterials, cephalosporins) use in Gansu, 2012–20. **c** J01F (macrolides, lincosamides and streptogramins) use in Gansu, 2012–20

Among of different antibiotic classes, J01C had the highest average use (14.96 DID, 45.15%). Its trend also increased first and then decreased, and the turning point took place in 2017. J01CA was dominant in penicillin use (13.77 DID, 91.45%). Although broad-spectrum penicillin use has gradually decreased in recent years, it is still the most used four-level subclass. In addition, the use of J01CR showed a slow-growth trend. The second most frequently used class of antibiotic was J01F (11.32 DID, 31.40%). Regarding its subclass, the proportion of J01FA (4.93 DID, 63.00%) was higher than that of J01FF (6.39 DID, 37.00%). J01FA has decreased significantly since 2014, and J01FF has changed slightly. J01D was the third most used antibiotic (3.97 DID, 11.99%), and its trend was the same as the total volume, but the decrease range was relatively small after the peak. The use of J01DB (1.75 DID, 43.34%), J01DC (1.13 DID, 28.38%) and J01DD (1.07 DID, 27.73%) decreased successively. J01DB use remained at a high level for several consecutive years. In addition to the above three antibiotics, other antibiotic use was relatively low (Fig. 2).

### Antibiotic use at public hospitals in Gansu, 2012–20

**Fig. 3:**
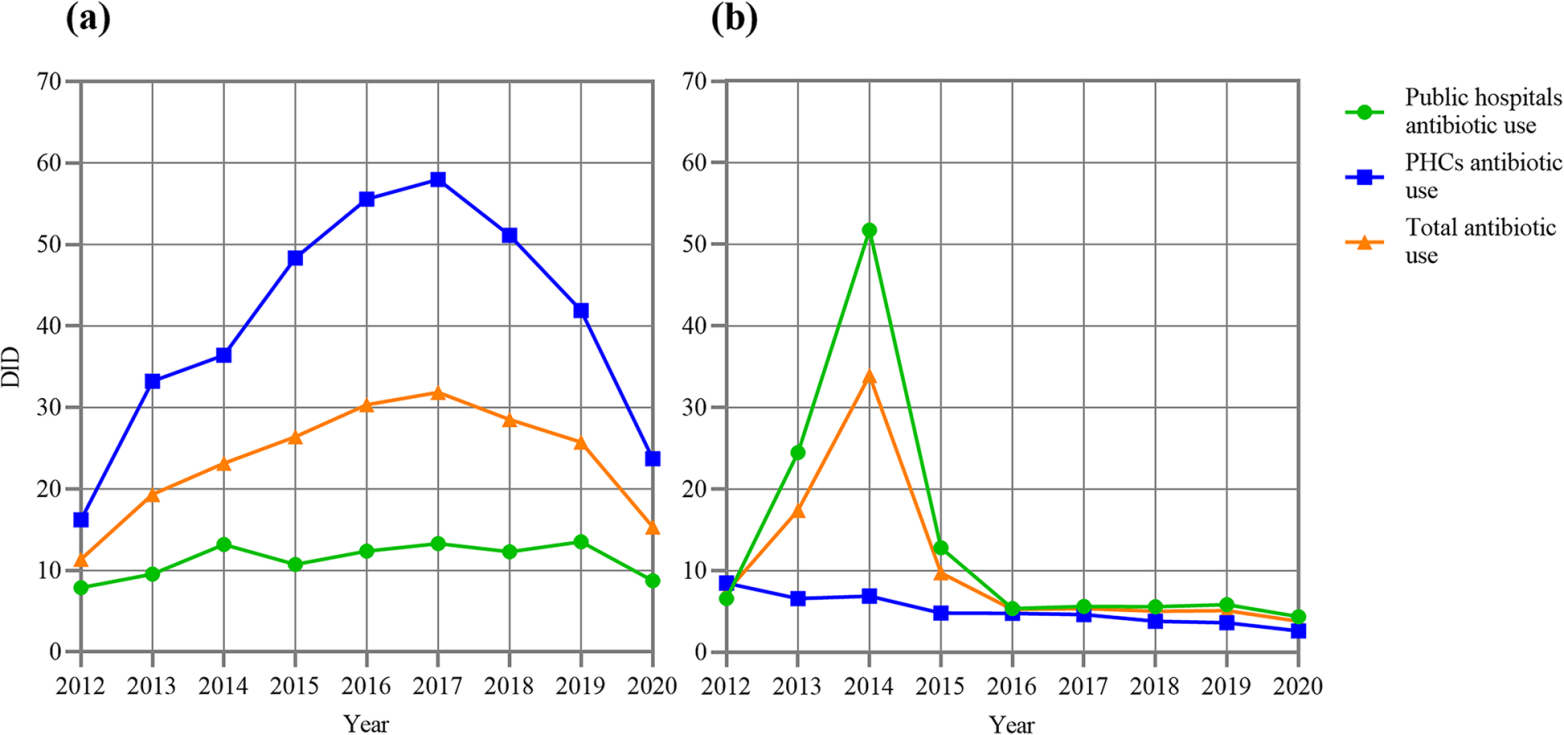
**a** Oral antibiotics use at public health care institutions in Gansu, 2012–20. **b** Parenteral antibiotics use at public health care institutions in Gansu, 2012–20

The changing trend of antibiotic use in public hospitals in Gansu Province from 2012 to 2020 was the same as the total, rising from 14.48 DID in 2012 to 64.91 DID in 2014 and then falling to 13.09 DID in 2020. The use increased the most in 2013 (134.85%) and decreased the most in 2015 (63.72%). The average use of oral antibiotics (11.28 DID, 54.89%) was similar to parenteral antibiotics (13.59 DID, 45.11%), and they shared a similar varying trend with that in the whole province (Fig. 3).

**Fig. 4:**
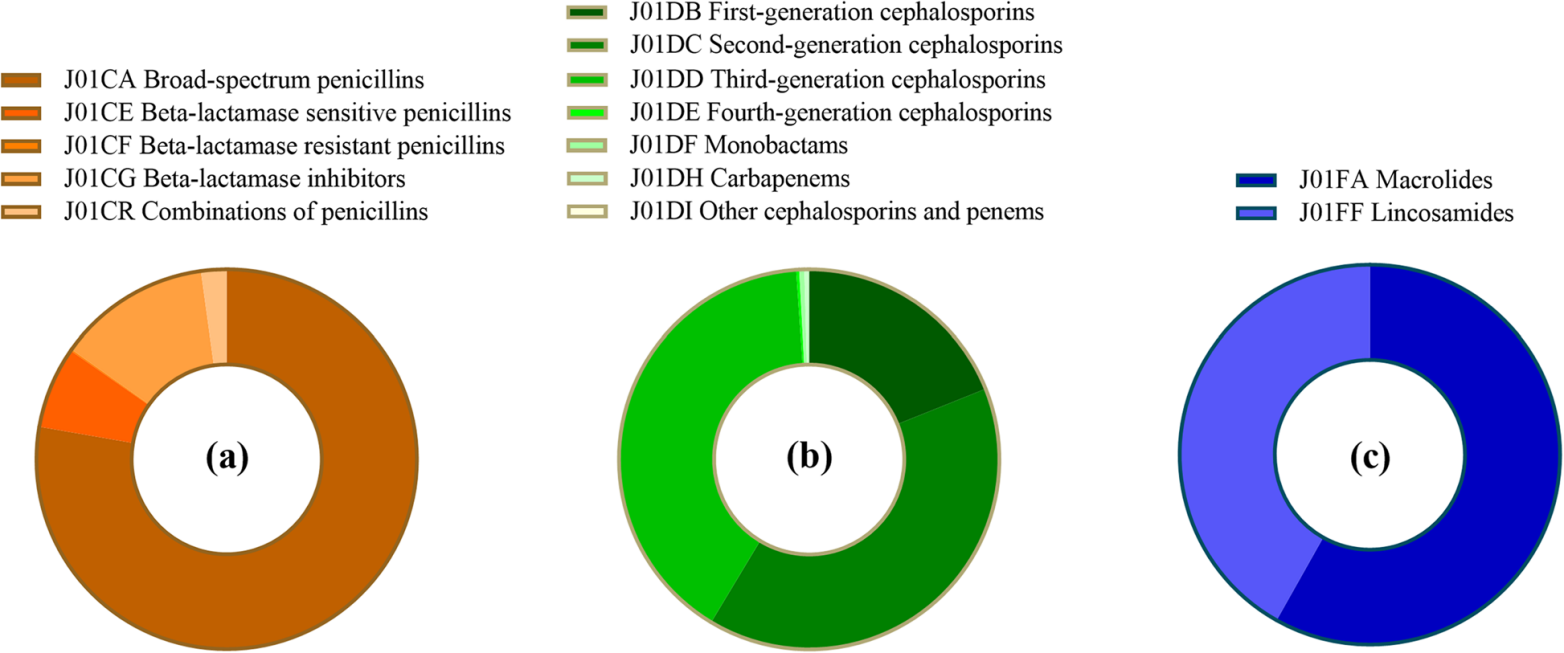
**a** J01C (beta-lactam antibacterials, penicillins) use at public hospitals in Gansu, 2012–20. **b** J01D (other beta-lactam antibacterials, cephalosporins) use at public hospitals in Gansu, 2012–20. **c** J01F (macrolides, lincosamides and streptogramins) use at public hospitals in Gansu, 2012–20

In public hospitals, J01F use was the highest (13.87 DID, 47.93%). Among its subclass, J01FF use was higher in the first four years, while J01FA use was higher in the last five years. This was followed by J01C (5.76 DID, 26.37%), of which J01CA (4.53 DID, 77.75%) accounted for the highest proportion. The third and fourth most commonly used antibiotics were J01D (2.95 DID, 13.92%) and J01M (1.36 DID, 7.03%) (Fig. 4). It is worth mentioning that J01DC (1.15 DID, 39.68%) and J01DD (1.22 DID, 40.30%) use was higher than J01DB use (0.55 DID, 18.93%).

### Antibiotic use at PHCs in Gansu, 2012–20

The peak time of antibiotic use at PHCs in Gansu was in 2017, where antibiotic use increased from 24.69 DID in 2012 to 62.60 DID in 2017 and then decreased to 26.32 DID in 2020. The use increased the most in 2013 (61.14%) and decreased the most in 2020 (42.17%). Oral antibiotic use (40.50 DID, 87.13%) was almost equal to the total (Fig. 3).

**Fig. 5:**
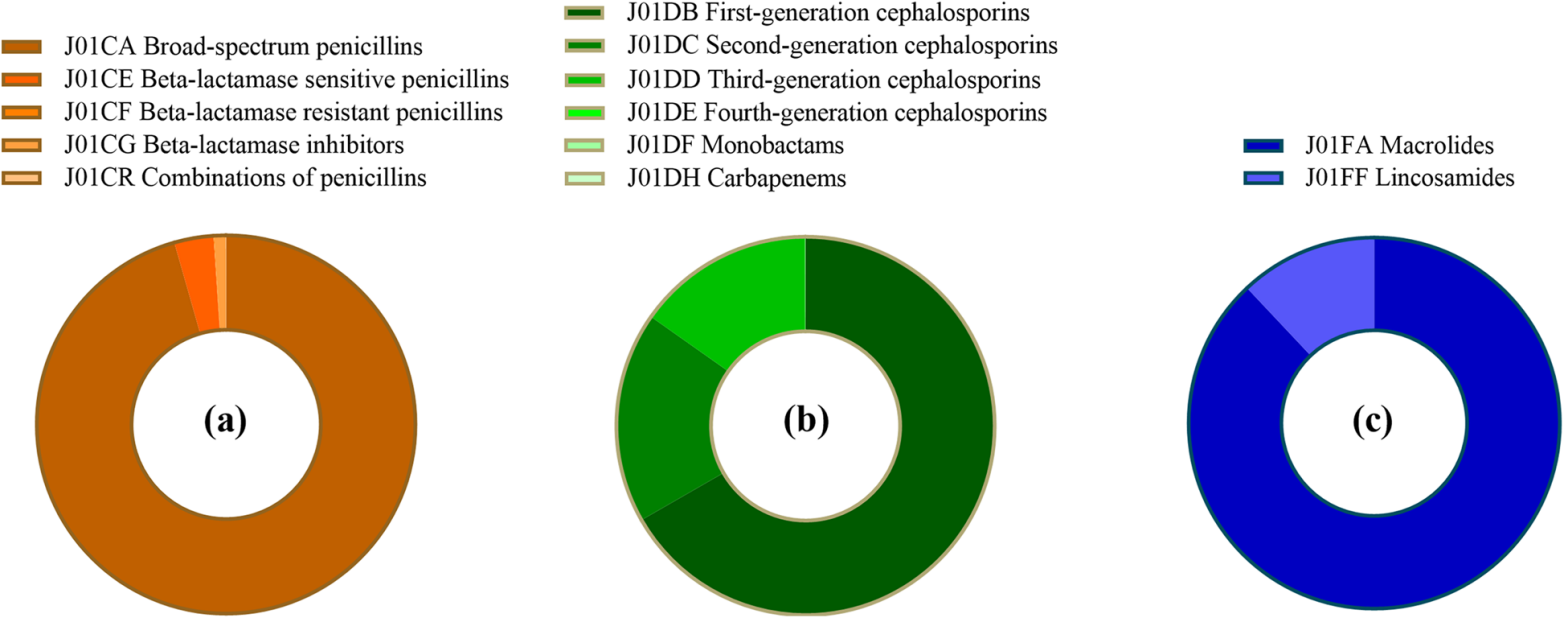
**a** J01C (beta-lactam antibacterials, penicillins) use at PHCs in Gansu, 2012–20. **b** J01D (other beta-lactam antibacterials, cephalosporins) use at PHCs in Gansu, 2012–20. **c** J01F use at PHCs (macrolides, lincosamides and streptogramins) in Gansu, 2012–20

J01C use (28.20 DID, 60.64%) accounted for more than half of the total use in PHCs, with J01CA (27.14 DID, 95.59%) being widely used. J01F use was the second highest (6.88 DID, 15.43%), and J01FA had the highest use (6.07 DID, 87.98%) among it (Fig. 5). This was followed by J01D (5.20 DID, 12.20%) and J01M (3.88 DID, 8.64%).

### Comparison with ESAC monitoring results

In comparison with the monitoring results of 31 European countries in the ESAC project in the same period, it was found that absolute antibiotic use in Gansu Province was at a high level, even higher than all countries included in the project for five consecutive years. This phenomenon was mainly reflected in J01C and J01F use, while the use of J01A and J01E was always low. In addition, although J01C use was high, J01CR use remained at a low level (Table 2).

**Table 2:**
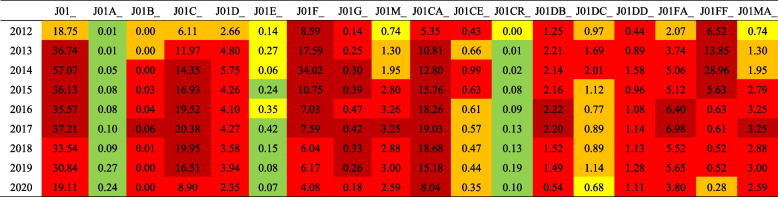
Comparison between total antibiotic use in Gansu and European countries, 2012–20

Antibiotic use in all monitored countries was arranged in ascending order.  Higher than all monitoring countries;  Higher than top 75% monitoring countries;  Higher than top 50% monitoring countries;  Higher than top 25% monitoring countries;  Lower than top 25% monitoring countries;  Lower than all monitoring countries

^a^J01_B/N: Ratio of the consumption of broad-spectrum (J01(CR + DC + DD + (F-FA01))) to the consumption of narrow-spectrum penicillins, cephalosporins and macrolides (J01(CE + DB + FA01));

^b^J01_SV: Seasonal variation of the total antibiotic consumption (J01). Overuse in the winter quarters (January-March and October-December) compared with the summer quarters (April-June and July–September) of a 1-year period starting in July and ending the next calendar year in June, expressed as percentage: [DDD per 1000 inhabitants and per day (winter quarters)/DDD per 1000 inhabitants and per day (summer quarters)-1] × 100;

^c^J01M_SV: Seasonal variation of quinolone consumption (J01M), calculation formula as above

Conversely, relative antibiotic use in Gansu Province was low. Especially regarding J01CR use and the ratio of broad/narrow-spectrum antibiotics, the results were lower than at least three-quarters of countries. The seasonal variation in total volume fluctuated greatly, but the seasonal variation in J01M was almost lower than that in all countries (Table 3).

**Table 3:**
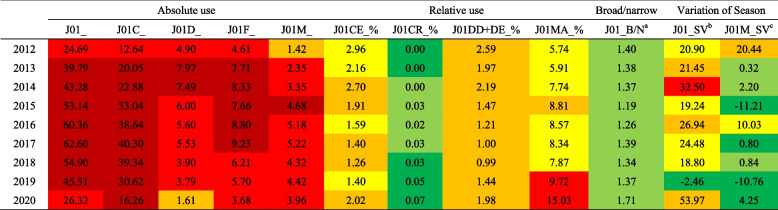
Comparison between PHCs of antibiotic use in Gansu and European countries, 2012–20

Antibiotic use in all monitored countries was arranged in ascending order.  Higher than all monitoring countries;  Higher than top 75% monitoring countries;  Higher than top 50% monitoring countries;  Higher than top 25% monitoring countries;  Lower than top 25% monitoring countries;  Lower than all monitoring countries

^a^J01_B/N: Ratio of the consumption of broad-spectrum (J01(CR + DC + DD + (F-FA01))) to the consumption of narrow-spectrum penicillins, cephalosporins and macrolides (J01(CE + DB + FA01));

^b^J01_SV: Seasonal variation of the total antibiotic consumption (J01). Overuse in the winter quarters (January-March and October-December) compared with the summer quarters (April-June and July–September) of a 1-year period starting in July and ending the next calendar year in June, expressed as percentage: [DDD per 1000 inhabitants and per day (winter quarters)/DDD per 1000 inhabitants and per day (summer quarters)-1] × 100;

^c^J01M_SV: Seasonal variation of quinolone consumption (J01M), calculation formula as above

## Discussion

A global action plan on antimicrobial resistance pointed out that high total antibiotic consumption was the main factor leading to the occurrence of AMR and was also the key to controlling the problem [29]. The current study showed that total antibiotic consumption in Gansu was higher than in some high-income countries and even higher than in France (37% higher) — the country with the highest antibiotic consumption in ESAC before 2017 [30]. There is no doubt that compared with high-income countries, the disease burden faced by early LMICs (mainly referred to as the burden of infectious disease) was much larger than the adverse effects caused by AMR (burden of AMR bacterial infection). Accelerated urbanization and socioeconomic growth are two mutually reinforcing conditions, followed by the spread of various bacterial infectious diseases and the prevalence of nonbacterial infectious diseases [31]. The former made a great contribution to the extensive use of antibiotics, while the latter was an important driving factor for unreasonable antibiotic use. In addition, the high sales were also related to the production, sale, and management mode of antibiotics in China. Compared with other high-income countries, Chinese doctors had fewer restrictions in issuing antibiotic prescriptions, patients had more channels to buy antibiotics, and the general public had a higher rate of self-medication with antibiotics [32, 33]. This phenomenon was also common in other LMICs in Asia [34].

After 2014, although broad-spectrum antibiotic consumption was still high, total antibiotic sales showed significant negative growth in Gansu, which benefited from a series of top-down policies and measures issued by China's health department. After the reform, the rapid development of animal husbandry exacerbated antibiotic abuse. At the same time, it also gave birth to China's first medicine management regulation, 'The Drug Management Law of the People's Republic of China', but the regulation was only controlled from the overall medical level [35]. After entering the twenty-first century, relevant policies dedicated to antibiotic use and management were issued. In 2012, the former Ministry of Health issued the "strictest antibiotic management method" — 'The Administrative Measures for the Clinical Use of Antibacterial Drugs'. The project lasted for three years. During this period, antibiotic consumption in public hospitals in many provinces and cities fell precipitously [36].

The study found that J01C ranked first in the consumption of various antibiotics in Gansu Province all year round, followed by J01F, which was different from the use patterns of other provinces and cities [37]. In China, there were some differences in antibiotic use patterns in the eastern, middle and western regions divided by geographical location. In the final analysis, the reason was that their regional environment, economic, social, and cultural backgrounds were different, and the distribution of medical resources and the population literacy rate were also very different [38]. These socioeconomic factors were directly or indirectly related to antibiotic use. The natural climate and socioeconomic factors were similar among the provinces (cities and autonomous regions) in northwest China, and the disease spectrum had some common features. Therefore, the antibiotic use pattern in Gansu province can represent northwest China to some extent.

J01F consumption in Gansu was at an abnormally high level before 2016, and the main contribution (83.89%) came from public hospitals. It has been suggested that this was mainly due to the widespread use of clindamycin which is sensitive to methicillin-resistant Staphylococcus aureus (macrolide-lincosamide-streptomycin B-resistant) in hospitals. The incidence of infections caused by this class of resistant bacteria was higher in hospitals than in the community [39]. This trend in J01F was similar to that in other regions in China [40]. In addition, the extensive use of broad-spectrum antibiotics was another concern. Although the consumption of broad-spectrum penicillins has decreased in recent years, it was still the most used subclass in J01C. Before the reform of public hospitals, driven by seemingly ideal treatment effects and higher medicine profit returns, clinicians often chose high-level antibiotics for treatment — high-grade rather than low-grade, broad-spectrum rather than narrow-spectrum antibiotics [41], which led to the fact that most antibiotics used in hospitals at that time were three or more generations, seriously violating the principle of antibiotic use. The super indications medication was also another important culprit leading to the increase of AMR [42]. 'The guidelines for the clinical use of antibacterial drugs' issued in 2015 clarified the basic principles of antimicrobial use and the treatment principles of multiple bacterial infections and emphasized the indications and precautions of various antibiotics. At the same time, related regulations also revised and improved requirements for management indicators and clinical application evaluation of antibiotics, stipulating that the number of antibiotics used in secondary and tertiary hospitals' antibiotic catalogues shall not exceed 35 and 50, respectively [43]. All these factors can explain the obvious decline in consumption of many subclass antibiotics after 2016.

The overuse of parenteral antibiotics cannot be ignored. The proportion of parenteral antibiotic use in Gansu decreased from 39.47% in 2012 to 19.88% in 2020, which was a great breakthrough, but the figure was still higher than the estimated 7% in European countries [44]. The main share of this part also came from public hospitals. A series of medical consumable costs attached to parenteral antibiotic use was an integral part of the medical cost, and some doctors may issue such prescriptions driven by interest [45]. Compared with oral antibiotics, patients often thought that parenteral antibiotics had faster, better effects, and shorter course of treatment than oral antibiotics. Therefore, they might flow to higher-level hospitals due to the demand for parenteral antibiotics [46].

The number of outpatient visits undertaken by PHCs in Gansu accounted for only 40%, and this proportion was as high as 80% in most European countries [47]. Compared with their strong primary health care service capacities, China's PHCs have been weak in this regard. To make matters worse, PHCs had more antibiotic consumption (53.93%) while having fewer outpatient visits. We adjusted the intensity of antibiotic use in PHCs according to the proportion of outpatient visits. It was found that the total antibiotic and penicillin consumption used in PHCs in Gansu almost exceeded all monitoring countries in the ESAC project, and other provinces also reported similar results [18]. As the implementation objects of earlier antibiotic application management policies were mainly concentrated in public hospitals and the constraints on PHCs were not reflected until a long time later, the use of antibiotics increased steadily before 2017. Moreover, most medical personnel in PHCs had not received unified and high-quality preemployment learning and training, resulting in a lack of professional knowledge and insufficient diagnosis and treatment levels in terms of antibiotic indications and incompatibility. Therefore, the phenomenon of empirical medication, preventive medication, and nonstandard medication is common [48].

To achieve a reasonable pattern of medical treatment, under the background of 'the guiding opinions on promoting the construction of hierarchical diagnosis and treatment system', Gansu decided to implement the graded diagnosis and treatment system of a new rural cooperative medical system in three pilot cities in 2014, it was fully implemented in the whole province after 2016 [49]. Objectively speaking, the system indeed played a certain role in reducing the use of antibiotics in PHCs, but it was still far from its original purpose of "clear division of labour in health care institutions and promoting reasonable medical treatment". The key to the problem was that PHCs lack reliable and excellent medical resources [50]. The shortages in medical workers, medical equipment, and medical medicine have not been solved, so the effect of correct diagnosis and treatment and rational drug use has not been achieved. Regarding the ratio of broad/narrow-spectrum antibiotics use, the ratio of PHCs in Gansu was better than in most countries monitored by ESAC, but this result should be interpreted with caution. This cannot be completely attributed to the fact that antibiotic use at PHCs in Gansu was more reasonable than that in other countries. The consumption of broad-spectrum antibiotics was maintained at a high level. Rational analysis suggests that it may be due to high consumption of total antibiotics, and identical high consumption of narrow-spectrum antibiotics will hedge the ratio, so it masked some of the real results.

In addition, this study still has the following limitations. First, the data used in the study were from procurement records, so they may not directly reflect actual antibiotic use. Second, the purchasing units included in the system were all public health care institutions, so the results cannot reflect the contribution of private health care institutions to antibiotic consumption. Third, as a province with a large population outflow, the actual resident population in Gansu Province may differ from the statistical population, and since the number of mobile individuals cannot be obtained and estimated, we use the mid-year population as the denominator when calculating the intensity of antibiotic use, which also leads to small calculation results to some extent.

## Conclusions

Based on the antibiotic purchase data of Gansu Province in the last nine years, taking different levels of health care institutions as the starting point, this study analysed antibiotic use patterns and long-term trends, and compared calculated quality indicators with ESAC monitoring results. It was found that antibiotic use in Gansu Province has decreased sharply since 2014, which was consistent with intervention policies issued by China's health department. The decline was most obvious in public hospitals and relatively slow in PHCs, so the follow-up control of antibiotic consumption should designate PHCs as the main target. The relatively large proportion of broad-spectrum antibiotics and the still high use of parenteral antibiotics are signs of irrational antibiotic use. Therefore, it is necessary to further standardize the quality of antibiotic-related prescriptions and pay particular attention to the rationality of antibiotic use in PHCs.

## Data Availability

The datasets used and/or analyzed during the current study are available from the corresponding author upon reasonable request.
